# A Longitudinal Goal Setting Model for Addressing Complex Personal Problems in Mental Health

**DOI:** 10.1145/3555160

**Published:** 2022-11-11

**Authors:** ELENA AGAPIE, PATRICIA A. AREÁN, GARY HSIEH, SEAN A. MUNSON

**Affiliations:** University of California, Irvine, USA; University of Washington, USA; University of Washington, USA; University of Washington, USA

**Keywords:** Human-centered computing, Empirical studies in HCI, HCI theory, concepts and models, Empirical studies in collaborative and social computing, mental health, goal setting, collaborative reflection

## Abstract

Goal setting is critical to achieving desired changes in life. Many technologies support defining and tracking progress toward goals, but these are just some parts of the process of setting and achieving goals. People want to set goals that are more complex than the ones supported through technology. Additionally, people use goal-setting technologies longitudinally, yet the understanding of how people’s goals evolve is still limited. We study the collaborative practices of mental health therapists and clients for longitudinally setting and working toward goals through semi-structured interviews with 11 clients and 7 therapists who practiced goal setting in their therapy sessions. Based on the results, we create the Longitudinal Goal Setting Model in mental health, a three-stage model. The model describes how clients and therapists select among multiple complex problems, simplify complex problems to specific goals, and adjust goals to help people address complex issues. Our findings show collaboration between clients and therapists can support transformative reflection practices that are difficult to achieve without the therapist, such as seeing problems through new perspectives, questioning and changing practices, or addressing avoided issues.

## 1 INTRODUCTION

Many people aspire and seek to improve themselves or change their behaviors. A crucial step in making progress towards behavior change or improvement is to set goals [45]. Setting goals is a component of effective health interventions that have behavioral components such as improving diet, exercising more, smoking cessation, or mental health therapy [1, 26, 34, 66]. Technologies can facilitate setting health behavior goals, like exercising more [22, 23, 44, 51]. Many of these technologies support quantitative goals, like the number of steps walked, number of calories consumed, number of hours of sleep. However, the goals people want to achieve can be qualitative and multifaceted [21, 55, 59]. Even though a person might set a goal of walking a number of steps, they might also have more abstract goals such as being more active, feeling good, or being a better person. The complexity of people’s goals has been surfaced in the context of managing health conditions when people set goals that need to account for the complexities of managing their conditions and to problem solve [48, 59]. People setting complex goals have found it effective to work with clinicians who collaboratively set goals with them [21, 48, 59, 60]. However, researchers still have a limited understanding of how to help people choose and adjust goals that are appropriate to their context, abilities, and needs, especially for complex or abstract goals. Further, people’s goals and needs can evolve over time [55]. For these reasons, practitioners can find it challenging to support people who need to set complex goals.

Mental health is particularly well-suited to studying complex goal setting. Decades of research in mental health demonstrate that effective evidence-based psychosocial therapies involve setting goals and planning out activities for the client [19, 37]. Setting goals and planning helps people engage in everyday activities that people typically disengage from during when depressed, and lowers depression [25]. Mental health researchers have found that mental health goals can be particularly difficult for clients to manage: people do not necessarily know how to address the problems, people might not recognize the problem right away, and the problems might change in significance or difficulty over time [26]. The process of scaffolding goal setting into therapy, and the collaboration between clients and therapists offer a unique window into the goal-setting process over time.

In this paper we studied goal setting in the context of mental health to understand how people longitudinally identify and modify goals, and what supports goal-setting behavior. Specifically we seek to understand the practices and lived experience of clients and therapists who collaboratively set goals to navigate complex situations and manage depression and anxiety. To understand goal-setting behavior, we interviewed 11 clients and 7 mental health therapists about how they set and modified goals over nine weeks of therapy sessions. Our work contributes:

1. Understanding of how goal setting manifests in mental health and the strategies used by clients and therapists to address complex issues the client is facing. Their strategies included: understanding the multiple multifaceted problems a client is faced with to select which problems to address, refining complex problems or goals to specific ones, and adjusting goals based on the client’s lived experiences. We find that clients and therapists go through a collaborative process of transformative reflection. This process involves the therapist gaining a deep understanding of the client, their problems, and their goals. The process can then support the client to challenge assumptions, gain new understandings, and change their practices and behavior.
2. A three-stage model characterizing how clients and therapists use transformative reflection of the client’s problems and lived experiences, to select, simplify, and adjust complex goals to meet people’s needs. Our model informs the design of technologies that support collaborative pursuing and setting goals.
3. A discussion that draws on collaboration practices used in mental health therapy to suggest how technology can support goal selection, simplification, and adjustment. We explore techniques such as collaborative reflection and leveraging other people’s expertise to re-envision or anticipate problems.

## 2 RELATED WORK

### 2.1 Goal setting in HCI

Research in HCI has surfaced ways to help people set and achieve goals. Systems like UbiFit [23], GoalPost [51], and Fish’N’Steps [44] enable people to set goals and track their progress toward them. These systems commonly encourage people to set goals of a daily step count (e.g., 10,000 steps per day as an ongoing goal), a set of weekly exercises, or a number of calories eaten in a given day. However, while many tracking technologies support goals that can be expressed in quantitative metrics, easily measured goals are not always representative of the goals people want to achieve [24]. Research shows that people have qualitative goals that they want to set—like being a better person or feeling good—that get transformed to goals that technology can support [55]. People’s goals also change over time [10, 20, 30]. People’s physical activity goals may change from qualitative to quantitative. For example, they may change from “feel well” to “lose weight,” and then to quantitative goals like “walking 12K steps per day” [55]. People’s tracking goals for migraine management change over time as they gain a better understanding of their health condition or as their symptoms and circumstances change [59, 60]. People managing chronic conditions sometimes change their goals by reflecting on different temporal frames of their collected data [48]. However, little is known about how people choose and adjust goals that fit their needs, the support they need in doing so, and how design and technology can better support goal choice and adjustment.

Although there is some understanding that people’s goals change over time, current technologies provide limited support for people to choose the right goals for themselves and later adjust them, particularly for complex goals. Research shows how technologies can support people in adapting goals to different difficulty to account for what might be achievable for a person. For example, tools might prompt the user to set a step goal as a minimum number of steps and the minimum number of days per week they would achieve it (e.g., “I will walk at least 8,000 steps per day on 3 or more days this week.”) [52], to set backup or alternate goals [20, 51], to set margins for their goals [6, 33], adjust the difficulty of the goals based on progress [43, 52], account for people’s situations when setting a goal [61, 62], or set specific actionable goals through planning [7, 8]. However, changing the difficulty of goals does not actually address whether the goal itself is appropriate for the person; it is possible that an entirely different framing of a goal might better support them. Individualized support can help a person adapt their goals by taking their preferences into account [7, 21] or encourage them to self-understand through experimentation [35, 36, 40]. For example, technology can provide scaffolding—support in which people get personalized help to create exercise plans that account for their preferences, constraints, and routines [7].

#### Reflective practices to support goal setting.

Reflection has been found to be an effective approach to help people set goals. Reflective activities can involve going through different phases of reflection. These phases can be categorized, according to Fleck and Fitzpatrick [32], as *descriptive reflection* (involves noticing actions and events, reasons for actions, but with no further analysis or change in perspective), *dialogic reflection* (relationships are drawn between events, interpretation and questioning occurs), and *transformative reflection* (challenging of personal assumptions, and further understanding leads to fundamental changes in practices and understanding). Reflection can support reframing and in-depth understanding of whether a goal is a good representation of what the person wants to achieve. Reflective practices of inquiry and transformation of perspective [11, 32] help people reflect on higher-level goals the person might have [18], or better understand their behavior and personal data [13].

Research in personal informatics shows that reflection can support people’s understanding their motivations for a goal, leading to revelations. Revelations help people devise better plans of action to reach their goals, and can improve goal setting and make future actions more feasible [38]. The Goal Tracker Evolution model shows that reflection is a necessary part of people’s understanding of their goals, particularly qualitative ones [55]. The Technology-Mediated Reflection Model explains how reflection occurs in people’s personal informatics cycle [12]. It shows that reflection assists practices such as understanding people’s data and progress over time, setting goals, relating data with abstract goals, and reflecting on their goals at different levels of abstraction [12].

Even though we know that people’s goals change, most of the research in the HCI community has focused on easily quantifiable goals, such as step counts, exercise, or screen time. These quantifiable goals make sense when the HCI community starts with what can be easily sensed and tracked with technology. However, these goals are too narrow when we compare them with goals that are most important in people’s lives. People also report that their goals change as they make progress, relapse, or their circumstances change. Research on reflective practices in HCI shows that reflection can help people better understand themselves, draw new insights and perspectives, and respond by taking new actions. But there is still limited knowledge on how people set complex goals. In this study, we address this gap in understanding of how people set and iterate on complex goals, by researching goal-setting practices used in mental health therapy, a context in which people set goals to address complex issues they are facing.

### 2.2 Technology supported goals for mental health

Over 25% of the US population is affected by mental illness [5]. Less than half of people who are facing mental health challenges access professional support [2]. Given people’s lack of access, many technologies have been emerging to support people in self-managing their mental health. The Apple and Google Play marketplaces contain an estimated over 10,000 apps for behavioral health [17]. Numerous apps advertise themselves as supporting goal setting. Such apps support the delivery of digital therapeutic interventions (e.g., Bloom CBT), self-care and wellbeing (e.g., Fabulous), and stress management (e.g., Headspace). These apps explicitly support people to set goals, or implicitly present goals as activities the person could do, such as taking a walk, eating mindfully, sleeping better, managing everyday anxiety and stress, being more productive, and decluttering. Such apps tend to take a more holistic approach to goal setting than other health or tracking apps. Apps support behavioral goals like exercise, diet, and sleep (e.g., Fitbit, MyFitnessPal). They either support people self-set goals or provide people with programs that show several steps through which people could make progress on a long-term goal. Apps that support people with multi-step programs thus enable them to set and work on a goal over time, check in with whether the user completed the goal, or offer suggestions about how the user could implement changes to reach the goal (e.g., Fabulous). Although wellbeing apps take a more holistic approach at supporting different aspects of people’s life, they support limited customization. That is, the apps present goal setting features similarly to all users, and limited customization to user context. Similarly, although they offer longitudinal support, apps offer only generic programs that might not account to the user’s change in goals.

While HCI research in mental health has increased, goal setting is not not a primary focus [58]. Research that can inform goal setting is situated in self-management of mental health, though understanding what type of support people need in mental health activities, but not through longitudinal goals. People desire customized support for mental health, but true customization still remains a challenge with technology [63]. Some approaches that can help people achieve their goals include self-generating personalized solutions to chart a path to goals, supporting self-managing mental health through reflection [42], and preparing them for addressing stressful events by anticipating future situations [41]. People can also receive customized support by connecting with others. When people connect with others, they can understand themselves better [42], receive empathy [14], and engage in social activities [14]. People can receive customized support from others who know them well [14] or through structured conversations with online peers that can help them see their situation differently [50, 56].

Even though technology has been used for mental health management, understanding how technology can support goal setting for mental health is still an open challenge. Given the increase in apps that support behavioral health, there is a need to understand how to support longitudinal engagement with goal setting for mental health through technology.

### 2.3 Therapy approaches for goal setting

A crucial element of evidence-based psychosocial interventions (e.g., Behavioral Activation, Cognitive Behavioral Therapy, Problem Solving Therapy, and Cognitive Processing Therapy) is goal setting [19, 37]. Approaches to goal setting vary across psychosocial interventions, but it is a common function of helping patients overcome obstacles to self-selected goals [37]. Goal setting in psychosocial interventions are commonly implemented by setting goals that are meaningful to the client, relevant to the central goals of therapy, are agreeable to both client and therapist, have clear rationales, and are appropriate to the sociocultural context of the client [65].

Evidence-based psychosocial interventions that are have been proven effective at lowering depression symptoms and have a goal-setting component include Problem Solving Therapy (PST) [26], Engage Therapy (ENG) [9], and Behavioral Activation (BA) [34]. These therapies use a multi-step approach to goal setting, in which clients and therapists set goals and create action plans. These steps are organized around therapy sessions (typically weekly) and work between sessions. Below we present the different steps involved in the process of goal setting across the different interventions:

1. Clients and therapists set a goal based on a problem that clients want to solve (e.g., “I eat too much sugar at night, and it affects my sleep”) (PST), or an activity in which they want to be more engaged (e.g., “I want to be more mindful about what I eat”) (ENG, BA)
2. Select a goal for the week that addresses the client’s problem or desired goal (e.g., “Only have on chocolate after dinner.”) (PST, ENG, BA)
3. Brainstorm strategies to meet the goal (e.g., “eat the chocolate very slowly, to enjoy it”, “tell my partner that I want them to support me in eating fewer sweets in the evening”) (PST, ENG). Then weigh the feasibility of each strategy and select the most feasible strategy (PST),
4. Create an action plan to implement the goal, and implement the action plan outside of therapy (e.g., “after cleaning dishes I will take one chocolate and leave it out”, “I will eat the chocolate after prepping lunch for tomorrow”) (PST, ENG),
5. Review how helpful it was and troubleshoot if it was not helpful (PST, ENG, BA). Although therapy manuals document best practices for therapy, there is limited research on how therapists apply goal setting in practice and the strategies they use.

We draw on the collaborative practices of clients and therapists in mental health practice to understand collaborative goal setting for complex mental health problems. These insights inform how technology can better support setting complex goals, as well as how it can support collaborative goal setting.

## 3 METHODS

### 3.1 Participant Recruitment

To understand the collaborative and lived experience of clients and therapists through goal setting during therapy sessions, we conducted interviews with clients and with therapists who practiced a therapy with a goal-based intervention: Problem Solving Therapy (PST), Engage Therapy (ENG), or Behavior Activation (BA). We recruited participants from 9-week programs in which the client and therapists would meet weekly and were practicing PST, ENG, or BA therapies. The programs we recruited from consisted of two randomized controlled studies [3, 4]. The programs provided training to therapists in delivering one of the PST, ENG, or BA therapies, and asked therapists to practice the therapy intervention during sessions with clients. The clients were already enrolled in the 9-week therapy programs, or had completed the program in the past two months. The studies targeted the delivery of therapy to older adults. The clients involved in the study were experiencing at least mild depression at the time of enrollment in the therapy programs, according to PHQ-9 assessments. Clients had participated in the programs for 3 to 9 weeks. We had IRB approval to recruit from the therapy programs described above and conduct interviews with participants.

We recruited 7 therapists practicing PST, ENG, and BA therapies. All therapists had been provided training in PST and ENG, were evaluated by experts in PST and ENG, and passed a quality standard of delivering the therapy according to expert evaluators. The primary occupations of the therapists were a social worker (T1, T3, T7), social work professors (T2, T5, T6), and therapist at a community mental health clinic (T4). The therapists stated that their practice involved Cognitive Behavioral Therapy (4), motivational interviewing (2), behavior activation (1), and that they also used other approaches such as psychodynamic (2), eclectic (1), DBT (1), solution-focused therapy (1). The therapists had received degrees in Master of Social Work (3), clinical social work (1), or participated in other post-graduate training (1). One interviewee did not disclose their degree. Most therapists had been practicing for the past one to five years, only one therapist practiced only for the duration of the therapy program from which we recruited.

Therapists practicing BA also had recent experience practicing PST and ENG, so their interviews were focused on the most recent therapies they practiced. Among therapists, we interviewed 5 about their PST practice and 2 about their ENG practice. The 7 therapists identified as male (1) and female (6), with ages that ranged from 26 to 54 years old (median age of 41). They identified their race as Caucasian/White (3), Asian (1), and African American/Black (2). We compensated therapists with gift cards of $50 per hour. Interviews lasted 60 to 90 minutes.

We recruited 11 clients (Table 1) who were receiving one of the therapy interventions: PST (4), ENG (4), or BA (3). Participants identified as male/man/M (6), female (4), or identified as human (1). Participants were between 61 and 81 years old. They identified their race as Caucasian/White (9), African American/Black (1), or Other (1). The clients were either actively participating in the 9-week therapy program, or had completed the program in the last months. That means participants had been in therapy for depression currently or in the past month. We compensated clients with gift cards of $25 per hour. Interviews lasted 60 to 90 minutes.

### 3.2 Interview approach

We structured the interview around an activity in which all but the first two participants walked through a grid in which they described their experiences of going through different steps of the therapy process. These steps included uncovering what issues to address, coming up with solutions or ideas to address the issue, identifying the next steps to address the issue (action plan), implementing an action plan, and reviewing the implemention. We organized the activity based on the best practices from therapy manuals about how a therapy session for PST, ENG, or BA would be organized. Although each therapy is different, we characterized the steps of goal setting in terms that would be applicable to all participants. We validated with participants whether the grid content was aligned with their experience with the therapy.

### 3.3 Data analysis

We used used a mix of inductive and deductive approaches to analyze the data and conceptualize themes. First, five researchers open coded two different transcripts, then in a meeting discussed the emergent codes and their meaning. The codes were organized in an affinity diagram based on similarity. The researchers developed a codebook based on the emergent codes and wrote code definitions. We continued coding using the codebook and having weekly meetings, between which everyone coded approximately two transcripts. The codebook was edited as questions or exceptions came up to more clearly define codes. We updated the codebook as new codes emerged through the remaining transcripts. The deductive approach to the codebook was to include codes that captured the challenges that clients and therapists encountered at different stages of the therapy process, based on the best practices of the therapy (e.g., discussing problems, setting goals, engaging in talk therapy, filling worksheets, and checking in on how the week went).

The five researchers had different data analysis tasks. One researcher was a coder for all therapist interviews, while a second researcher was a coder on all client interviews. Two other researchers coded transcripts, and each transcript was coded by two different researchers. The lead researcher also contributed to the coding, read all the transcripts after the coding process, and added codes as needed. Three researchers wrote memos as they analyzed the data. During the weekly meetings, we discussed codes and themes in the data. We did not conduct inter-rater reliability, as any misalignments in coding supported our process of synthesizing data, understanding themes, and revising memos [47].

## 4 RESULTS

We found that clients and therapists work together on addressing client goals, that span multiple aspects of their life. The client and therapist engaged in transformative reflective practices that helped the client identify goals that could help them navigate difficult situations, problems, or goals they strove towards in their everyday life. We identified three major steps in setting goals. First, clients and therapists selected a problem or goal that they planned to address together, potentially from multiple multifaceted problems. Then clients and therapists simplified the problem or goal they chose to a specific version that could be addressed through concrete steps by the client. Last, after the client experimented with working towards their goal, the client and therapist assessed whether they needed to adjust the goal or move on to a different issue. Given the nature of the context we are analyzing (therapy sessions), the types of goals discussed were complex and difficult for clients to achieve. Participants used strategies to turn complex goals into goals that clients were more likely to achieve. Throughout the goal setting, we highlight points of reflection that the client and therapist engaged in: noticing aspects of the client’s life and goals, drawing understanding of the client’s goals and life, building connections between insights, and transforming understanding through changing perspectives about the client problems or effective approaches to addressing goals. These elements highlight a process of *transformative reflection* that clients and therapists go through at different stages.

### 4.1 Mental health goals set across multiple aspects of life

Participants set and worked towards a variety of goals, targeting problems in their life that changed over time. Below we describe the range of participant goals, problems, and the extent to which people transitioned between different goals. We describe the processes of goal setting and goal evolution in more detail in the next sections.

#### 4.1.1 Types of goals set.

Participants in the study set goals that addressed situations they perceived as undesirable: a problem. Clients encountered situations that they wanted to change or improve, which were referred to as “problems” during therapy sessions (e.g. improving relationships, managing grief, reconnecting with people). We will use the terminology of problems to be consistent with the participant language. A problem orientation is consistent with the therapies in our study, which use goal setting to address problems in people’s lives or to address disengagement from regular activities through setting goals.

When they joined the therapy program, several clients felt depressed and did not know what to do to feel better. Clients characterized this state in terms of feeling “stuck” and not feeling like engaging in activities in their everyday life: *“No, I’m not getting up”* (C6), *“I just don’t feel like doing anything”* (C5), *“I needed to be still”* (C8), or *“I was stuck, and when you’re stuck it’s just easy to stay there”* (C7). Clients wanted to do something to change the state they were in, feel better, and *“get out of the rut”* (C8).

Participants had multiple different problems that they wanted to address at any given time: *“sometimes you may have four or five things”* (C6). All participants set multiple goals as part of therapy sessions over the nine sessions they attended. The types of goals that people set, or problems they tried to address ranged from improving relationships with their spouse, adult children, or relatives (6); connecting with other people to address isolation (3); doing more pleasurable activities (4); managing the death of a partner, child, or family member (4); being more physically active (3); losing a job (1); managing finances (3); decluttering their homes (3); and managing their own or family member’s health issues (4). A client explained how the original issues they wanted to work on were: *“I don’t socialize. Health. My lymphedema, lose 10 pounds. Colonoscopy . . . home de-clutter . . . because when my husband died”* (C3). Therapists acknowledged that people’s issues span many aspects of a person’s life. In addition to therapy, therapists also helped clients connect to other resources (T1, T4) to address basic needs, like filling in appropriate applications for housing (T4) or to access transportation (T1). Clients corroborated this. For example, C5 received resources from their therapist about managing sleep, how to clean out the condo, or guidelines for how to store important documents.

#### 4.1.2 Goal changes over time.

Participants changed their goals over time in several different ways. Therapists took multiple sessions to get a sense of what problems the client was encountering: *“by the third session, I have a pretty good sense of some core problems that we can work on based on their problem list or based on what they keep bringing up”* (T5). In addition, participants changed what issues they were working on. For example, C1 switched from a goal of improving her relationship with her daughter-in-law (3 weeks) to managing her relationship with her son (on and off 5 weeks), C3 switched from discussing issues related to her husband’s death to goals that helped her socialize (e.g. going to an exercise class, going to church, going to a genealogical group), C4 switched between the weeks from managing communication with his wife, to planning activities with his wife, to trying to be less isolated through visiting the senior center, C5 varied his goals between exercising, doing archery, and cleaning his home, and C6 switched goals between being more physically active to improving relationships and connecting with others. Other participants focused on a primary problem like trying to find a job (C2), engaging in more pleasurable activities while managing his wife’s illness (C10) or managing grief for son’s loss (C8), recovering from loss of family (C11), and reconnecting with people (C7). Even participants who focused on one predominant issue chose to set different goals at times.

Other clients felt they could only work on one issue at a time: *“sometimes you may have four or five things and I personally don’t believe you can do it justice if you take more than one thing at a time”* (C6). Although during therapy sessions the clients and therapists set one primary goal every week, some clients revisited other problems in their life outside of their therapy sessions *“She [therapist] always has me pick a new issue or a new problem to work on. And that way most of them at least get addressed whether I resolve them or not. But I’ve taken it upon myself to go back and work on problems that aren’t yet resolved”* (C4).

In the next sections, we describe how clients and therapists identified goals by understanding and reinterpreting client experiences, and problems (Table 2). They also identified goals based on prioritizing problems.

### 4.2 Selecting among multiple problems and goals

Clients and therapists collaborated on what goal to focus on at any given time. Clients had a multitude of problems that they wanted to address, and identified goals to address their problems in collaboration with the therapist. Choosing what goal to focus on involved identifying what goal was most relevant based on current issues, discussing past problems, and anticipating future challenges.

#### 4.2.1 Accounting for multiple problems across different aspects of life.

Therapists and clients identified what to focus on during therapy sessions by starting with a list of problems that the client wanted to address (T1, T4, T5, T6, T7). This approach was based on the therapy process they used, which recommended creating such a list in the first session of therapy. The problem list included multiple aspects of life, such as health, social, or work. This list was not static. Therapists added problems that they and the client identified to an ongoing problem list over time (T4, T6). Clients found therapist help useful in identifying problems: *“[therapist] helped me identify several areas in my life that I wanted to work on, so that I could reengage with life and feel better about the future.”* (C7).

Some therapists expected clients to change how they perceived problems, which could lead to new problems emerging, or other problems disappearing from the list over time: *“they had all these problems, and they’re like these things suck, but once you start feeling better, you’re like oh yeah, I accepted that a long time ago that my sister and I are never gonna be friends, so that’s really not a problem”* (T5). The client and therapists first engaged in *descriptive reflection*, through which they noticed patterns of the client’s behavior. They recognized patterns by noticing aspects of the client’s behavior from conversations, or through reflecting on tracked data. Afterwards they would engage in *dialogic reflection*, by identifying relationships between current behaviors, values, mood, to inform the next actions the client could take to address their goals.

*Problems that are under the client’s control.* Portions of the multifaceted problems clients encountered were under the control of the client, and others which were not. Situations that were identified as out of the control of the person included problems like chronic pain or lack of housing. The client and therapist engaged in *dialogic reflection* by interpreting whether situations were under the control of the client or not.

When the client did not have control over the problem, the therapists encouraged setting goals that helped manage and change things about that situation, for example a client who had undesirable housing focused on what they could improve about it: *“how do we make this a place that still nurtures and still got a roof over our head... “But my bed is really uncomfortable.” So that’s the problem solving we did”* (T3). At other times, the therapist guided the client towards emotionally managing the situation. For example, a client had a problem they did not feel like they had any control over *“Kids stopped talking to her, refused to let her see her grandkids. . . . She had no control. . . she couldn’t change this certain situation. Now what we worked on a lot was stress management, things she could control”* (T1).

*Choosing goals aligned with client values.* An approach therapists and clients used to select between multiple goals was to choose based on client values. Throughout this activity, the client and therapist engaged in dialogic reflection, understanding relationships between the client’s values and behavior.

Some therapists asked participants to identify values that guided their life during the therapy session. Some clients reflected on whether tracked data aligned with their values helped them decide which goal they wanted to set. For example, C6’s values were *“having open communication . . . mutual respect; spending time with my siblings”*. Every week the clients would choose goals aligned with these values, along with activities that made her feel better in the past: *“it’s called a values inventory. . . we set up activities. And then . . . I actually put those activities into motion... and then we discuss that and how that felt”*.

#### 4.2.2 Revisiting unresolved or avoided problems and goals.

Both clients and therapists discussed how it took time and iteration to discover client problems. While sometimes clients would directly articulate the problems they wanted to resolve, at other times they took time to surface. The client needed to resolve a different issue before moving on, or they only felt ready to take on a new problem after addressing a prior one. At other times, problems were revisited because the client was avoiding working on them. At this stage, therapists and clients engaged in *dialogic reflection*, trying to understand relationships between the client’s different problems and behavior. Therapists might challenge the client to engage in *transformative reflection*, by bringing up problems that the client was avoiding.

##### Revisiting avoided problems.

Some clients avoided topics that were difficult to discuss because the client did not feel ready to address them, were avoiding them, were not sure if they could bring them up, or they were embarrassed about them. For example, C6 set goals to communicate better with his wife by setting a goal to *“learn to start conversations with her”*. However, he avoided confronting the situation. The therapist noticed that he avoided this for six weeks, and brought it up: *“he feels abused by his wife... that’s a big part of his life and... a lot of his depression... he has problems in the other categories. Like loneliness or feeling too sedentary or his diet... And he always picks those, but then it feels we’re talking around the bigger issue of, ‘Should I leave my wife or not?’”* (T4).

Therapists tracked the different issues in a client’s life through a problem list, developed in the first session, or as the client brought them up. For example, T5 tracked their client’s core problems based on how often they brought them up. However, the client did not always want to set a goal related to the core problem: *“they might say this is a problem. . . or my marriage sucks. . . but instead, she wants to focus in on her exercise plan because she’s feeling like she has some extra pounds when it’s pretty clear we can focus in on her marriage”*. Some clients did not bring up important problems, because they were difficult to address (C1). T5 periodically brought up problems that were previously stated but not addressed: *“It’s not like I pushed it, but... if you keep bringing up a problem to me, I will kind of bring it up again”*. In other cases, clients were not sure if they could bring up certain topics. C2 did not know if he could talk about his relationship with his sister, because the primary focus of the sessions had been on helping him get a job. C10 did not feel comfortable bringing up things that he thought were embarrassing.

##### Transitioning to goals that the client felt ready to address.

Several clients took two or three sessions to talk about a difficult problem they were facing *“we sort of climbed up this little mountain and then we got to the top and said, ‘Oh, here’s the real problem’”* (C1). One client (C1), discovered a new problem that occurred after first working on a different problem in her life. Working on her relationship with her daughter-in-law surfaced the need to work on a more important relationship with her son. C3 needed help managing her feeling around her husband’s death, which readied her to move on to addressing other problems *“me socializing, me going out and doing exercise class, me going to church, me going to genealogical meetings, getting out and being social”* (C3).

#### 4.2.3 Anticipating future challenging situations assisted by therapist external perspective.

Therapists were attentive and reactive to their clients’ situations. When a therapist noticed that an undesirable situation was approaching that the client would have difficulties navigating, they would bring it up and work with the client to find an approach to navigate it. The reflection on the different situations that the client might be facing, suggests that the therapist might be engaging in *dialogic reflection*. This reflection emerged through conversations about what was currently happening in a person’s life.

When the therapists and clients surfaced a potentially difficult situation, they came up with a goal that would improve it. For example, a client’s source of joy was her boyfriend. When the client and therapist learned that he would travel, keeping them apart, they tried to identify other activities that could bring the client joy: *“he’s leaving town. I’m really anxious, and then we say... this is an important thing for us to prepare for. . . so we’re trying to figure out alternate activities that bring the same joy”* (T1).

In some cases, the client and therapists worked for several weeks on managing a future difficult situation. C3 anticipated that attending her granddaughter’s graduation would be stressful due to the potentially difficult interactions with another family member. She and the therapist spent a few weeks coming up with a plan for how to manage the event, such as whom to sit next to, and whom to avoid during the event. Their planning shows that client’s goals sometimes were set based on urgent issues emerging in their life, instead of working on longitudinal or more important issues (C6).

### 4.3 Simplifying complex goals

Client problems were representative of the complex goals that participants wanted to achieve. Problems were multifaceted, broad, and changed over time, making it hard to immediately identify a goal that would address them. To identify actionable ways to address problems, clients and therapists went through a process of understanding the different aspects of problems. This process scoped the problem to one that was specific enough to be addressed through goal setting. Understanding problems occurred through breaking down problems into different components. Throughout this process, the therapist contributed new perspectives, which led to the clients seeing their situations through a different lens.

#### 4.3.1 Understanding different aspects of problems.

Clients and therapists engaged in *descriptive* and *dialogic* reflection by understanding the different aspects of a problem and how they manifested, then breaking them down into smaller issues. They drew connections between different aspects of a problem to better understand them and identified actionable steps towards resolving them.

##### Understanding how problems manifest.

Depression and anxiety can manifest in different ways in a person’s life. To know how to address a person’s situation, therapists needed to understand how a person’s mental health challenges manifest in their everyday life. For example they understood what the client does when they feel anxious or depressed: *“I tend to go in my bedroom and lay on my bed. And then I realize that I’ve been on my bed for several hours”* (T3). The therapist connected this information to insights they gathered about what the client likes to do and then identified a potential goal and plan of action with them: *“they’ve mentioned that they really enjoy walking, gardening, being outside, things like that. So we’ll talk about, ‘...what could you do instead of going into your room and laying down?’”* (T3).

##### Breaking down problems to specific goals.

Some of the client problems were difficult to immediately translate into a goal. To identify goals, clients and therapists identified multiple sub-problems: *“You have to drill down and determine just what those problems are. Each one of those can lead to two or three”* (C4). It took time to address the different sub-problems in several therapy sessions: *“some of those relationship problems could eat up three sessions”* (T5). Breaking down problems felt like growth and was empowering for C1 to manage the problem, who: *“became more powerful . . . I would be able to see the problem and oh, okay this. . . It’s not that huge. So we can begin to dismantle it”* (C1). As the client and therapist collaboratively broke down problems, the number of issues they had to address can become larger, which could lead them to again select a problem to focus on.

Participants broke down complex problems into more specific ones, which they would translate into an actionable goal that was easier to address. Therapists encouraged clients to generate ideas of how to address the problem or to speak in detail about how they will take action to address the problem: ‘What are you going to do when you... What are you going to do about it this week? When can you fit that into your schedule?’” (T4). Therapists suggested approaches for addressing a problem based on their knowledge of the client.

##### Understanding problems as a longitudinal process.

Breaking down complex goals was a longitudinal process for many clients. For C1, breaking down problems was essential for her journey to address her relationship with her son, and that it took time: *“to break something down into parts helps to get the problem solved rather than this big gorilla in the room. . . it’s something that will take time. It’s not something that it’s going to [be resolved], if I talk to him once”* (C1). Some of the situations that people wanted to change had been bothering them for a long time. For example, reconnecting with friends was difficult after going through a long period disconnected: *“I felt anxious and resistant, due to the length of time I had been detached from life, friends, family, and community”* (C7). Another aspect of the longitudinal nature of the issues addressed was the permanent presence of the issues in their life. C1’s relationship with her son was challenging: *“he’s always omnipresent on my back... Like a backpack. I have to carry him around with me... And always tied to the past, always.*” C3 needed more than the length of the therapy program to resolve her feelings around her husband’s loss, *“nine weeks is not enough time to talk about anything like that*.” A therapist explained that it can take nine weeks to address one significant problem a person has before moving on to something else: *“We’re only meeting for 50 minutes... So not all the problems are going to come out, and not all the things that we should be more insightful about are going to come out. . ., I think that you could do this for nine weeks, and then you could start over for nine weeks targeting something else”* (T3).

##### Challenges in breaking down problems.

Understanding different aspects of a problem was a difficult for issues such as maintaining a relationship, and managing grief or pain. When clients and therapists were not able to collaboratively decompose large problems into smaller, addressable ones, they sometimes selected problems that they were perceived as less meaningful. C11 wanted to address *“Guilt around the death of my family members”*, but neither he nor the therapist knew how to address this issue: *“I don’t know what else I can do about my feelings of guilt about my family. . . I was clueless as to how to break it down into tasks, and so was she”*. This challenge led to addressing a less important problem: *“I talked about the fact that... I don’t have a libido anymore. One of the things that I miss in my life, some degree of intimacy.”* The client went on to set a goal related to finding information that could help him on this topic, even though he did not find it as important. Understanding problems across a person’s life was also overwhelming for some clients and therapists, because it surfaced too many problems (C4, C6): *“you can have so many problems that the therapist looks at them and goes, ‘Oh, my God. Where do we start?’”* (C4).

#### 4.3.2 Reinterpreting problems through therapist-driven inquiry.

Over time clients saw problems from a different perspective, which led to changing their goals. This change in perspective was assisted by the therapist, who challenged the client to see problems from a difference perspective, thus engaging in *transformative* reflection. For example, C3’s problem evolved from wanting to learn how to deal with family to not needing family as a support system, to wanting the family to treat her as an adult: *“I wanted to learn how to deal with the family better. And then by the time I left, what I decided is I don’t need to use my family as my main support system and that, I need to get them to treat me like an adult... It’s me treating myself as an adult and not using them as a crutch”* (C3).

C10’s therapist helped him see his problems through a different lens, which surfaced something he had not noticed before. C10 described this process as *“she brings up saying . . . have you thought about it like this? Have you thought about it like that? How does that hinder you from doing your list or getting stuff done or moving forward in your life?”* (C10). In this process the therapist surfaced that *“it sounds like you’re letting your wife’s illness be a barrier.”* This realization was a significant moment for him in thinking about how he was addressing his problems: *“it just went boom. Just in that succinct little sentence. . . well I’ve known that all along but I’ve never heard it that way. . . That’s one of the things I was hoping. . . to come in and get some kind of aha or clarification. And I did”* (C10).

Therapists listened to what the clients brought up, and sometimes identified problems and reflected that to the client: *“I’ll say, ‘This is a relationship you’re interested in rebuilding,’ and I can frame it in a present-tense way and put it on the problem list”* (T4). However, if a client had a problem that she did not know how to address, she would avoid discussing that problem with the client out of fear of not knowing how to help them: *“We should brainstorm solutions to that. But I have to think of a problem that we can brainstorm solutions to, and if I can’t think of any options for myself then I don’t want to put that on the client to do”* (T4).

#### 4.3.3 Identifying feasible goals through understanding and interpreting tracked data.

Clients commonly set a goal to *“feel better”*, without knowing what they could do to reach that outcome. This was particularly the case for clients, who were no longer engaging in activities that they used to enjoy, a common consequence of depression. Clients and therapists collaborated on a goal to help the client feel better through engaging in pleasurable activities. If a client already had an activity they wanted to do, they would continue with that goal. Clients and therapists needed to identify what enjoyable activities the client might want to do, and then use those activities as goals. However, several clients found it difficult to identify an activity (C3, C5, C7, C8). To translate the broad goal of “feeling better” to concrete pleasurable activities, the client tracked data and reflected on activities and mood in collaboration with the therapist (dialogic reflection). Based on the insights, clients drew conclusions that changed their perspective about themselves (transformative reflection).

To identify goals, the client tracked activities that made them feel good throughout the day, even hourly. By tracking their activities, clients could choose the ones they felt good about, thereby setting goals that continued to make them feel good. This was a typical approach of people who practiced Behavioral Activation as a therapeutic technique. During the therapy session, the client and therapists discussed and interpreted the tracked data, drew new understandings and insights, which led to new practices for the client.

Participants experienced transformative moments that revealed new insights and perspectives on their own life, which had a large impact on them (*“brilliant”* - C9) or a revelation of the things that were going well in their life: *“I had lots of aha moments like I didn’t realize I was doing that [activities] right”* (C6). C6 and C9 monitored activities and mood, which enabled them to see new things: *“This forced me to categorize things, and I look at it and go, ‘Oh, my god. That was a really good day’. I think I was in a mental fog. This helped break through it”* (C9). C9 learned the types of things that made her feel good through monitoring: *“I had moments of wonder, and fun, and meaning from things that were usually associated with other people, friends or family, or I went to see some fabulous movie, or rent some book that I loved.”* The pleasurable activities that surfaced then became potential targets for setting goals.

A therapist used activity monitoring to facilitate their client’s reflection, see relationships between their actions and how they feel, and finally to surface goals that the client could set: *“she noticed when she brought in her first two daily monitoring forms for the first two weeks, she was like, There’s no walking, there’s no physical activity. She just really was feeling like I got to change this... but the daily monitoring really brought it to her attention”* (T1). The monitored data was interpreted in collaboration with the therapist (C6, C9). C9 mentioned that she would have not been able to interpret the data without discussion with their therapist: *“She helped me get back to who I was in the discussion. Just doing it on paper wouldn’t have done it without the discussion”* (C9).

### 4.4 Adjusting goals

Over time, therapists and clients updated and changed goals when the client was not successful at achieving what they set out to do. Adjusting goals was guided by the therapist, who tried to understand the client’s skills in completing a variety of goals, understanding and anticipating barriers, and reflecting on current goals and practices to understand what could be helpful for the client.

#### 4.4.1 Understanding client skills and learning new skills to enable goal adjustment.

Therapists sought to understand their client’s skills to help them make progress towards their goal. This was achieved through reflection on the different aspects of the client’s behavior (descriptive and dialogic reflection). The client experimented with new skills, which sometimes led to transformative reflection, as they realized new ways of approaching a situation. Therapists took the time to learn about what a client can accomplish through experimentation and observing them for a few weeks. They observed how the client engaged with the goals they set in therapy: *“So it really takes a few sessions before you kind of get a sense of what a person is capable of . . . whether or not they finish their action plans and how well they finish them. Did they not do it at all? Did they do part of it? Did they do it, instead of going and walking twice a week, they went walking three times a week kind of thing”* (T3).

Therapists helped frame goals based on their clients’ skills. Sometimes therapists taught clients new skills, like how to have a difficult conversation: *“a lot of them [clients] are taking steps to do something maybe you’ve never done before. . . a woman, she had never told anyone that she was mad at them.... her whole life, been passive, and never said a word to somebody like I’m mad at you”* (T5). C3 described how learning skills allowed her to see her situation from a different perspective: *“Because I was getting better in doing things on my own, then the ideas changed, my needs [changed]”* (C3).

C1 described the transformational experience of seeing her situation as a transition between focusing on her son, to focusing on herself by learning and applying new skills. C1 transformed her view of herself as an *“inadequate mother”* to her adult son, to seeing detrimental aspects of her relationship with him, and finally shifting her need to *“extracting myself from the oppression”*. C1 addressed this problem by learning new ways to communicate with her son in different contexts. First, she learned how to handle situations in which her son *“goes after me verbally”: “I try to talk to him adult to adult. . . and then I try to stay very calm... I’ve learned how to say, ‘We can continue this conversation if you’re not yelling’”*. C1 also learned how to handle other aspects of her relationship with her son. She stopped herself in the moment from picking up after him around the house, and instead communicated with him *“even though I see my coffee cups on the bathroom counter. . . I have to learn how to say, ‘we’re running short of coffee cups. Would you bring those down from your room and the bathroom?’”* When her son used her car repeatedly without permission, she drew a contract for him to pay for using the car.

#### 4.4.2 Anticipating and reflecting on barriers.

Therapists wanted to help clients accomplish their goals by anticipating what barriers might come in the way (dialogic reflection). Therapists did so by drawing connections to prior knowledge they have about what goals are achievable for different people. If a goal was too ambitious, the therapist was concerned that their client would not achieve the goal and feel like they failed. Some therapists could anticipate that a certain goal will be too ambitious for the client. In such cases, some therapists had conversations with clients to try to elicit whether the client thinks they will be able to pursue a particular goal: *“Sometimes people want to plan out a lot when you as a therapist know that that might be too much. . . . then the question becomes... ‘Really, can you do this in a week before you see me? What part of that do you think you can do?’ So not setting them up to fail, or allowing them to set themselves up to fail”* (T3). Elicitation sometimes focused on client’s past experiences with goal pursuit. Some clients changed their goals by recognizing barriers that came in the way of their goals through reflecting on their behavior: *“I use her illness as my own barrier to get stuff done. I kind of use that as a reason why not to do something”*(C10).

#### 4.4.3 Reinterpreting current goals and practices.

Therapists drew on the client’s prior experiences to inquire about the client’s assumptions in setting goals to address problems. This adjusted the therapists’ approach to making progress towards a goal, and as a result, changed it. In the cases we identified, the inquiry was driven by the therapist. Clients were involved in experimenting with different goals and approaches, or reflecting on their approaches outside of therapy. The inquiry process led to client’s uncovering new perspectives (tranformational reflection).

A therapist questioned a client’s goals of being more physically active. The client repeatedly set a goal of going to yoga, which they did not achieve. While the client persisted in setting the same goal, the therapist questioned this approach and encouraged setting a different goal: *“insisted on going to a yoga class, and by week seven, he had yet to make a yoga class. . . during our time together he was getting more active. But he could not get to a yoga class. And finally, I was like, ‘Does it have to be yoga? Maybe it’s kickboxing or something else’”* (T3).

The therapists helped drive inquiry on the client practices when they felt like they were not helping the client. A therapist discussed how a client wanted to improve her sleep but she was concerned about taking her sleeping pills because it would cause her to snore and wake up her husband. The therapist questioned the client’s underlying beliefs, and triggered a different course of action: *“it was this belief that they had to be in the bed together, otherwise it was a sign that the relationship was not good. And so we questioned where those beliefs came from and whether or not it was a bad thing if she took a sleeping pill. . . She took her sleeping pills. She and her husband had a conversation. . . If it bothered him, he’d get up and go to another room”* (T3).

Therapists encouraged questioning practices by facilitating the client’s reflection outside of therapy, which led to the client changing their perception of what goals were helpful. Their reflection sometimes required therapist assistance. A client re-considered activities that were fun for her, after the therapist challenged her to do so *“she’s telling me that my fun activities are not fun . . . and she’s making me think about what I’m doing”* (C3). After reflecting, she no longer saw the pleasurable activity she was engaged in as *“fun”*: *“I had to write a paper and so [doing] researching... And so this is not a fun activity . . . we started working on my fun activities, like a manicure, pedicure is a fun activity. Going to get a cup of coffee with your girlfriend”* (C3). These activities were not easy to generate for the client, and took time: *“It took me a week to write that list. You know, most people... jot down 10 things are really fun to do”* (C3).

### 4.5 Developing a Longitudinal Goal-Setting model for addressing mental health problems

**Fig. 1.**
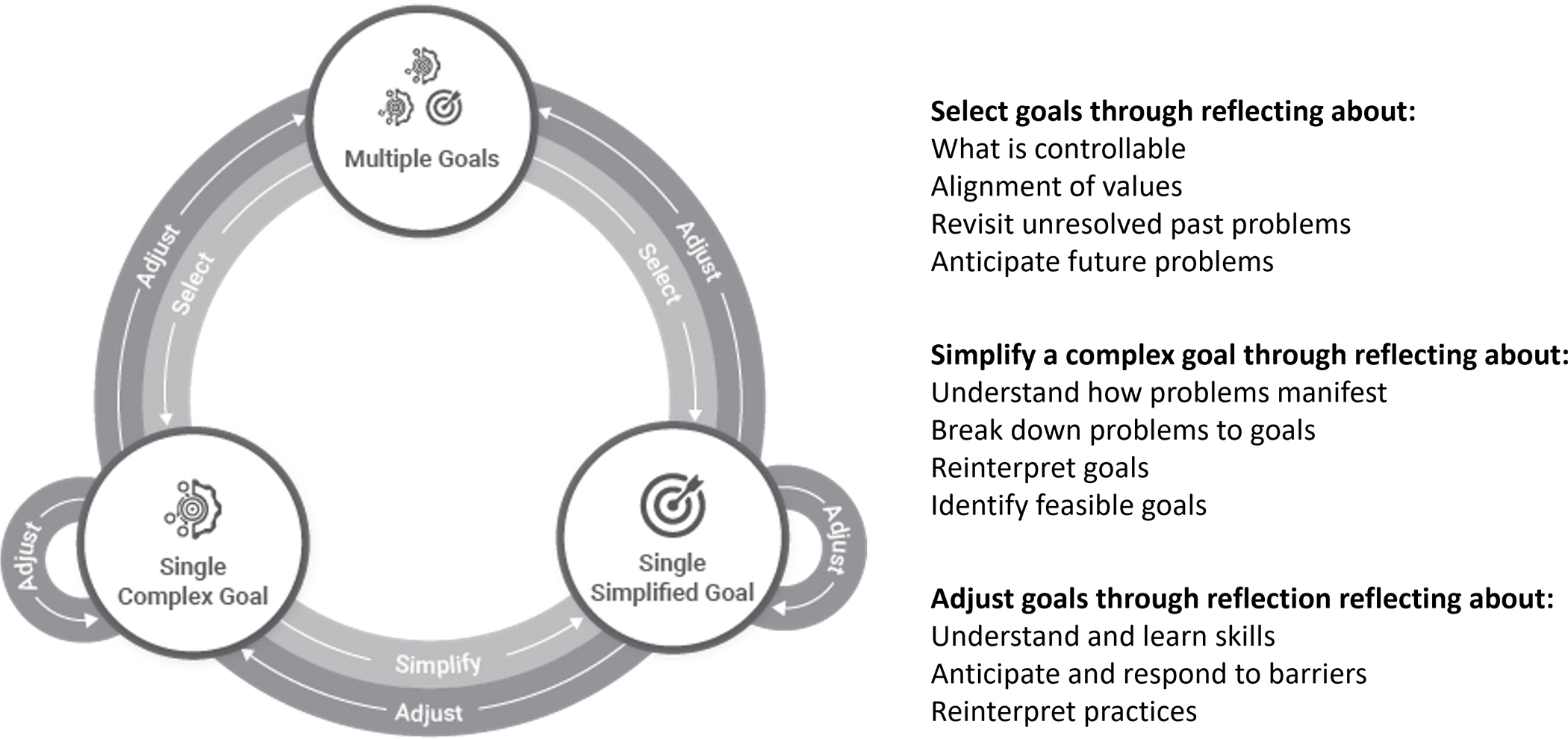
The Longitudinal Goal Setting Model Stages: Select (between multiple complex goals), Simplify (complex goals to a simplified goals), Adjust (simplified or complex goal to fit person’s needs better). At each stage, the process of Selection, Simplification and Adjustment involve a mixture of desriptive, dialogic, and transformative reflection.

Based on our findings, we propose the Longitudinal goal-setting model (Figure 1) that describes the practices involved in goal setting in the context of mental health. The model shows how people set, simplify, and adjust goals while working on complex problems they are faced with. Underlying this model are therapeutic practices that guided the structure of goal setting (Problem Solving Therapy, Behavioral Activation). Therefore, the steps observed in this process are rooted in therapy practice, and in how the therapists and clients enacted therapeutic practices.

#### 4.5.1 Stage 1: Select.

The *Selection* phase of goal setting refers to identifying which issue the person will address. Some people might not know which problems or goals they want to address. To help decide, people can select a problem that they have control over changing, and that is aligned with their values. The process of selection involves reflecting on and reinterpreting the present problems the person has, revisiting past problems, and anticipating future ones. In the context of mental health, people’s problems might span multiple aspects of their life, so it is important to account for the variability of problems the person might be faced within the selection process.

Selection among past problems or goals involves understanding problems that a person might be avoiding or was not ready to address in the past. People might feel ready to address an issue after completing a past problem. Addressing problems avoided in the past can be particularly difficult, and it can beneficial to have assistance to revisit these avoided problems.

Selection can involve anticipating challenging situations that might come up, and that a person might want to be prepared to address. Anticipating a challenging situation benefited from an external perspective to bring attention to upcoming challenges that the person might not think to address.

#### 4.5.2 Stage 2: Simplify.

The Simplify phase of goal setting in mental health refers to scoping a complex goal or problem to a specific one. This can be done through understanding how problems manifest in people’s lives and decomposing them into a set of simpler problems or goals. At times, the problems need to be reinterpreted from a different perspective. Once a sufficiently specific problem is identified, people can identify an approach that they could use to address the problem and set that as a goal. When people do not know what goal they could set, tracking activities and mood can help them reflect and interpret what activities are helpful.

The simplify stage can be a longitudinal process. People can develop understanding about their problems and good approaches to address them over several weeks. During this time, the person can work on goals that help them understand the problem and possible courses of action. People can benefit from support by gaining a new perspective on what their problems are or how they could be broken down. Support can be best provided by someone with expertise in breaking down problems, such as the therapist.

#### 4.5.3 Stage 3: Adjust.

The Adjust phase of goal setting in mental health refers to adjusting a goal after it has been set. Adjustment can be done after the person tried the goal but has not achieved it. Techniques for adjusting goals include accounting for the current skill of the person (do they know how to do the tasks that would involve achieving the goal), accounting for barriers encountered in the past, or anticipating potential barriers. People benefit from the perspective of an expert who has experience with common barriers that other people encounter and can anticipate them.

Adjusting goals can also occur by learning new skills, which can enable new approaches for addressing a problem. Adjust goals can also be done through reinterpreting the goals set and the practices involved in working towards the goal. This can require recognizing that something might not be working, questioning the approach, and seeing a new way of making progress towards the goal. Individuals can have difficulty noticing when something is not working or with identifying new ways of doing things once they do, and so here too they can benefit from an outside perspective.

#### 4.5.4 Role of reflection.

The process of goal setting is deeply collaborative. In our study context, clients were assisted by the therapist to reflect on their goals, problems, practices, skills, barriers and the different approaches they took to selecting, refining, and adjusting goals. The therapist brought expertise about the goal-setting process, knowledge of common challenges that people encounter and how to address them, as well as an outsider’s perspective. All these skills were used to notice client behaviors (descriptive reflection), understand connections between problems (dialogic reflection) and reinterpret the client problems (transformative reflection). This led to a deeper understanding of the client’s issues and new courses of action. The way reflective practices are used for goal setting, according to the levels of reflection described by Fleck and Fitzpatrick (Fleck and Fitzpatrick 2010) are encountered as follows:

**Descriptive reflection** occurs when people track activities to identify what was helping them throughout the week, or when they shared how their problems manifested. This was typically done by the clients tracking data on worksheets, or sharing facts about their experiences.

**Dialogic reflection** occurs throughout the entire process and requires guidance. The therapist helped turning the Descriptive reflection into Dialogic reflection by adding interpretation to the tracked data, or by providing prompts to the client to reflect on outside the therapy. As clients mentioned, the therapist was essential to understanding their tracked data, showing the importance of collaborative reflection for achieving deeper levels of understanding of one’s experiences. The therapist guided the therapy process and strove to understand client problems and how to help the client. Their efforts resulted in constant inquiry, drawing connections, and understanding the client’s problems.

**Transformative reflection** can occur over time, as clients gain a deeper understanding of their problems. They go through potentially several cycles of selecting problems, breaking them down, setting goals, trying them, and adjusting. The transformative experience was often described through revelations that came up in conversations with the therapist, who inquired about the client’s practices or perceptions of their problems. Transformative reflection was aided by the client’s experimentation with attempting to reach a goal or tracking data.

#### 4.5.5 Longitudinal process: transitioning between stages over time.

People’s goals evolve over time, at different paces. Some goals that require deeper understanding or experimentation can change over many weeks; others can change through reflective practices in the moment. People might focus on one or more goals, and they might disengage and re-engage with the goal over time. People prioritize goals by reflecting on past, present, and future situations, which makes it possible to transition to a new goal or attend to a situation. As this prioritization takes place in a longitudinal process, people might be working towards addressing a particular problem with similar or different goals across multiple weeks. Working towards a problem over time can transform that problem into a simpler one, but it can also turn it into a more complex problem that is more difficult to address. People might go through this cycle repeatedly until a goal is addressed, or concurrently work towards different goals.

Transitioning between the Select, Simplify and Adjust stages can occur in any order. If people already have a problem they want to address, they could start at the simplification stage. If they already have a goal but they are not able to achieve it, they could start at the adjustment stage. Given that each stage involves reflection on people’s goals and practices, starting at any of the stages could also lead to transitioning to another (e.g., adjusting a goal might reveal problems that inhibit addressing the goal, which might lead to selecting a new goal).

#### 4.5.6 Considerations in using the Longitudinal Goal Setting model.

The Longitudinal Goal-Setting Model is driven by practices used in behavioral mental health therapies (e.g. Behavioral Activation, Problem Solving Therapy). The practices we identified at each stage of goal setting are not comprehensive, and other practices might be helpful to people as they go through each stage. This model is derived from a variety of contexts and goals (e.g., people who set goals to improve relationships, exercise, diet, finances, communication approaches, work practices). It captures the process used for reaching complex goals in the context of mental health. We anticipate that this model would facilitate designing and delivering supports for people who are having difficulty achieving a specific goal within one domain as well, by providing guides to support iteration on goals over time.

## 5 DISCUSSION

Our proposed Longitudinal Goal-Setting Model offers insights into the different processes involved in setting goals to address complex issues that might occur in a person’s life. We provide insights about how people arrive at goals through selecting from a variety of problems in a person’s life, simplifying the complex problems to specific goals, and adjusting them based on a person’s needs, over time. We discuss insights into how the findings add to existing HCI knowledge on how to support people in addressing complex goals, how technology can better support different stages of goal setting, and how collaboration can support goal-setting processes.

### 5.1 Expanding understanding of goal setting for addressing complex problems

The Longitudinal Goal-Setting Model in mental health explains and expands our understanding of how goals are set over time to address complex problems, with a focus on understanding of the processes involved in reaching complex goals. The model stages describe areas of opportunity based on the processes of goal setting in mental health, and more broadly to support people’s wellbeing goals in complex situations.

#### 5.1.1 The Longitudinal Goal-Setting Model in relation to other models.

We expand on previous models—the Tracker Goal Evolution Model [55] and the Technology-Mediated Reflection Model (TMRM) [12]—that were developed primarily from a self-tracking perspective. This expansion adds to designers’ understanding of the strategies people use to reach complex goals.

Expanding on Niess and Wozniak’s Tracker Goal Evolution model [55], the Longitudinal Goal-Setting Model in mental health adds to our understanding of how people select, simplify, and adjust personal goals over time. The Tracker Goal Evolution model highlights goal evolution between abstract and concrete. It highlights the role of reflection, particularly as goals evolve from the eudaimonic, hedonic, and qualitative to more concrete. The Longitudinal Goal-Setting Model enriches our understanding of the transition from abstract to concrete goals, through the reflective process that occurs throughout the selection, simplification, and adjustment stages. It also adds specific strategies used for reflection, and assistance provided by the therapist (e.g., inquiry, external perspectives).

The reflective stage in the Tracker Goal Evolution model relates closely to the TMRM model. We add a further understanding about the processes involved in reflecting on goal evolution. TMRM illustrates that people want to reflect on data at different conceptual levels. For example, the TMRM differentiates between reflections at low construal levels (e.g., walking 10000 steps) and high construal levels (e.g., “spending time to relax”). The TMRM model explains that technology in the fitness setting does not successfully link insights from tracked data to abstract concepts. In the Longitudinal Goal-Setting Model, reflecting between different levels of abstraction is particularly present in the Simplify and Adjustment stages. In other words, when people reflect on how to break down problems and goals, they turn abstract goals into concrete ones. People could also reinterpret goals, which could lead to transforming an abstract goal to another abstract goal (e.g., “wanting to treat family better” transforming into “wanting to not rely on family as much”). The Adjust stage involves reflection that transforms concrete goals to other concrete goals (e.g., instead of going to a yoga class, aiming to go to a job). The model also shows that by, adjusting concrete goals, people surface abstract problems that need to be addressed (e.g., questioning if one needed to sleep in the same room as their partner for managing a sleep condition).

We believe the processes identified in this study, which inform the Longitudinal Goal-Setting Model, likely occur in the tracking settings that informed the Tracker Goal Evolution Model and the TMRM. However, the conversational, expert-facilitated setting in which we conducted this research made these processes more observable in our work than in this previous research. Our results highlight strategies not surfaced in this previous work, such as inquiry-based reflection to transform goals and external perspectives (in this case, of the therapist). These strategies present new ways of seeing client’s problems, anticipating future problems, or receiving encouragement to revisit unresolved ones. The Longitudinal Goal-Setting Model also offers insights into how people manage complex goals. It expands our understanding about the evolution of exercise-related goals to a broader range of topics involved in mental health.

### 5.2 Reflection practices for collaborative goal setting

The client and therapists in this study went through a process of reflection about client problems, needs, and experiences. Reflective practices are discussed in several models in HCI literature [11, 12, 32]. Through the Longitudinal Goal-Setting Model, we add to how people’s collaborative reflection on their complex problems and context manifests through transformational reflection.

Reflection research in HCI has highlighted the importance of understanding and designing for inquiry and transformative experiences [11, 32]. The empirical understanding of how clients and therapists collaborate illustrates the lived experience of engaging in reflective activities. The lived experience of reflection through goal setting in mental health shows how Inquiry [11] results in understanding and reinterpretating aspects of a person’s problems. The reflection techniques used during therapy sessions illustrate how Descriptive, Dialogic, and Transformative reflection [11, 32] occur in practice through noticing past information, understanding different aspects of a problem, reframing issues, or reflecting on experimentation. The differences in reflection levels show that deeper levels of reflection were achieved only in collaboration, and were difficult to achieve by the client on their own through worksheets. One of the approaches that deepened levels of reflection was therapist inquiry. The approaches used in therapy can serve as a starting point for designers of sociotechnical systems to support people in transformative reflection. We describe opportunities for supporting reflection in the sections below.

### 5.3 Supporting selection among multiple evolving multifaceted goals

#### 5.3.1 Technology ecosystems that support multiple evolving goals.

Clients and therapists prioritized a multitude of problems facing the client. Some clients focused on multiple problems at a time, while others paused addressing a problem to attend to a temporary problem. Engagement with different goals and problems evolved over time. These insights have implications for how to support people in engaging with multiple goals over time from a holistic perspective, which is useful to better support health management [57].

The current ecosystem of technologies provides limited coordination between people’s data, despite a push to create platforms that aggregate health data [28]. A lack of data aggregation can make it difficult to support coordination across people’s goals, because people use multiple tools to manage their mental health [15]. For example, a person might use an exercise app to manage physical activity, and a sleep app to improve sleep patterns. Based on our insights, one of these problems (e.g., sleep) might take a higher priority in a person’s life, and lead to the other goal (e.g., exercise) being a lower priority. Disengagement from the exercise goal might be recorded as a lapse in current technology and could make the person feel like they are lapsing at their goals. Using a holistic perspective, technology could support people in engaging with the goals that are important to them, while acknowledging that they will not all have continuity. Integration between different goals could improve reflection on [67] and management of problems. While there is a push in integrating personal health data in personal informatics, the focus has largely approached aggregation as a technical problem rather than a design problem [67]. While our field recognizes the potential for integrating the delivery of mental health interventions across a suite of mental health apps [49], more research is needed about how to integrate support across applications for customized goal setting, to integrate people’s multiple, evolving goals, with varying levels of continuity and engagement across disparate domains.

#### 5.3.2 Supporting selection through collaborative reflection on past, present, and future problems.

To identify a goal, people had to select a problem they would address, among the numerous they were faced with. Supporting this process involved reflecting on the current needs (e.g., current goals or problems), anticipating if any future events can create challenging situations that could be addressed now, or assessing the readiness of the person to address unresolved problems. People can be supported by having tools that keep track of and easily prioritize their ongoing problems.

The therapists inquired every session about what might be helpful for clients to focus on. While clients sometimes knew the answer, at other times they benefited from the process of inquiry of the therapist, who helped anticipate future events that may be challenging for the person. The therapist plays a role in bringing up past problems that the client is avoiding working on. By learning from such practices, technology designers of goal-setting technologies can incorporate features that are not only past or present-focused, but also future-centric [41]. Temporal awareness of people’s life suggests a need for awareness of what is upcoming in a person’s schedule or life to help identify potentially challenging events or experiences. Our results show that tasks like choosing between problems can be overwhelming. A therapist could provide empathy and support, understanding a person’s problems and breaking them down to arrive at an actionable goal. Designs could incorporate support from selected others who could be supportive participants as a person navigates their problems.

### 5.4 Assisted goal simplification

An increasing number of technologies support goal setting for mental wellbeing (e.g., Fabulous, Bloom CBT) or for other specific health goals like exercising or managing sleep (e.g., Fitbit). However, they predominantly identify goals that the person might want or let them set their own goals. Apps then provide support for how one might accomplish that goal, often through tracking. The goals supported through technology are often quantitative. Mental health apps provide more support to help people set qualitative goals. For example, apps might support choosing from qualitative goals, such as improving relationships with family members, or general goals like reducing stress. However, what is commonly missing are approaches to making such goals more concrete. Technologies that support planning [7] can help people translate their goals into concrete steps, but that is harder to do if the user does not know how to address a problem.

The problems people wanted to address for their mental health were not immediately translatable into concrete steps. This suggests a need for tools to support people in better understanding and deconstructing their problems. In personal informatics reflective technologies, much of the data that people can reflect on is quantitative and does not always match the level of abstraction that people want to reflect on [12]. Clients and therapists reflected on the client’s experiences and abstract problems, indicating a need for representing data through technology in ways that might be more experiential and abstract. To support abstraction, tools can have predefined goals that are associated with sub-goals. Goal hierarchies are starting to be incorporated into technologies such as Dailyo (e.g., the goal of “focus on family” is accompanied by subgoals such as “family projects” and “family mealtime”). Such goals might help, but they are not customized to the needs of the individual and their specific situation.

People might not know how to break down complex problems on their own, and require assistance. Advances in technology show promise in implementing reflection through conversational agents [38] that could help people address their problems. However, some of the roles that the therapist plays are harder to replace with a tool. The conversations with the therapist move from descriptive reflection, knowing the facts about the client’s problem, to dialogic reflection that leads to deeper understanding, and finally to transformative reflection that can help the person see their problems with a new perspective. Prior research has identified conversational and collaborative approaches that can help people reach transformative reflection through guided prompts that encourage them to consider their physical activity through different perspectives and experiences [38]. It is still an open question how to design such conversational interfaces to support transformative reflection in other domains. Another approach that has helped people see their problems in a different light is leveraging the perspectives of peers. This approach might be particularly well-suited to mental health: our participants found it overwhelming at times to reflect on their problems. Having emotional support from another person could make the emotional experience easier. In several collaborative technologies, peers are guided through technology scaffolding in using prompts to inquire and help another person reframe their thoughts and see their situation from a different perspective [50, 56].

Even when people are supported by an expert such as a therapist, identifying specific goals can be difficult. Some goals are challenging enough that the therapist can also need help to identify the best approach. Therapists might benefit from systems that connect experts, who share techniques about how they help patients to address different types of problems.

### 5.5 Supporting goal adjustment through evolving multifaceted goals

#### 5.5.1 Supporting collaboration for goal adjustment.

Although prior research has identified ways to update a goal so that it is better aligned with people’s constraints or abilities [6, 33, 43, 51], this work has typically focused on adjusting the difficulty of the goal. By applying reflective practices, technologies could better help people set goals that are most relevant to addressing their problems, and adjust their goals over time. Prior research has called for more support of reflection as part of mental health technologies [53, 58], but reflection support is still limited. Collaborative reflection is useful in planning care in collaborative clinical settings [46]. Our results demonstrate some approaches that could further support goal adjustment, such as capturing barriers to working towards a goal and reflecting on how to address them. Clients might benefit from tools that support easy, in-the-moment capture of barriers to facilitate later reflection, either alone or in conversation with the therapist. Another helpful approach is to have tools that make it easy to surface potential barriers to a goal while adjusting it. Tools could help the client and therapist during the session move past barriers that the client encountered before and might come up again, or that other people have encountered in similar situations. Such tools could help the client and therapist make more effective decisions at the moment and develop contingency plans for barriers the client might later encounter.

A challenge for adjusting complex goals is knowing the client’s skills, or teaching them so that the client can have adequate approaches to address their goal. It can take weeks of experimentation for the therapist to learn a client’s skills. Therapists and clients could be aided by tools that can help people practice a variety of skills, particularly because they work on a wide range of problems. While some skills can be taught by the therapist (e.g., how to have difficult conversations with others), there are skills that might be better suited to other resources (e.g., how to apply for unemployment).

People who are seeing a therapist can get help with goal adjustment from their therapist. However, it is common that everyday barriers emerge that could not be anticipated during therapy. For such situations, clients can benefit from tools that can help them respond to the barriers as they encounter them. Such tools might involve techniques for addressing common or individualized barriers or how to adjust goals when the barrier cannot be readily addressed.

#### 5.5.2 Supporting transitions and updates in user status.

Some clients and therapists kept an evolving problem list. This list would get updated over time with new problems, because problem framing might change and some problems could disappear. Problems would arise outside of the tracked list through identification of developing situations, or after reaching transition points when a problem was sufficiently addressed to move on. Problems were not necessarily resolved, but different parts were addressed over time, and engagement with a problem could re-occur. Past research has shown that there is a need to support people in transition periods, either during disruptions or non-routines [16, 54], transitioning goals, after people lapse in using tracking technologies [29, 31, 55], or as they change motivations in using technology or engaging in specific behaviors [27]. People can go through different states in a depression journey, depending on how their symptoms vary [39], or as they manage other health conditions [64]. Our results show opportune moments for supporting transitions between different goals: when problems get addressed as people feel ready to take on a new problem or goal, or when a difficult situation is anticipated. This indicates that supporting transitions might involve a reflective process on past problems, assessing current priorities, and a future-centric approach [41] to anticipating situations.

Another aspect of supporting transitions is to understand how perceptions of the client on their problems or goals change. Participants mentioned that problems might cease to be seen as problems, or that things that are perceived as problems might go unstated. Similar challenges in needing to keep an updated user status were found in prior research that showed that people’s preferences and constraints in how they pursue goals change over time [7]. Our results indicate that systems should regularly reassess people’s perception of their problems and goals for a more accurate representation of user priorities.

## 6 LIMITATIONS

The participants in our study were recruited from 9-session mental health programs in which therapists were asked to practice specific types of therapies (PST, ENG, BA). The way any individual therapist conducts therapy in their practice might therefore have been different. The clients were compensated to participate in the 9-session mental health program, so they had high motivation to engage with their goals. Clients with less motivation might exhibit different patterns in their longitudinal goal setting and pursuit. The population recruited for the study were older adults who experienced depression. We do not know how our findings of goal setting and evolution may translate outside of this population. The therapies practiced by the participants are focused on behavioral goals (people engaging in activities). Other therapies can also include goals that are cognitive in nature (e.g., reframing thoughts). Setting, updating, and pursuing cognitive goals might involve different processes.

## 7 CONCLUSION

In this article we have presented a model of Longitudinal Goal Setting in mental health. We identified three stages of goal setting: selection, simplification, and adjustment of problems and goals through reflective practices. This model captures the practices of mental health therapists and clients in setting goals. It offers a better understanding of how people set goals to address complex problems, the processes they use collaboratively in setting goals, and helps surface opportunities for technology to better support longitudinal goal setting.

## Figures and Tables

**Table 1.** Participants (id in study, self-reported age, gender, occupation, summary of issues addressed during therapy sessions).

ID	Age	Gender	Occupation	Issues addressed through goal setting	Other major issues related to goal
C1	70	Female	Retired	Manage relationship with son, manage the relationship with sister-in-law, Resolving paper-work	Relationship with her son is not good
C2	61	Man	Retired	Visit unemployment office, Apply for job	Lost job and forced into retirement, feels he was tricked and should be able to collect unemployment, not able to find work
C3	63	Female	n/a	Be more social, manage finances, manage the loss of husband, be physically active	Stopped being social after husband’s suicide, not feeling motivated, has difficult relationships with some family member
C4	70	Male	Mech Engr Technician	Improve communication with his wife, do pleasurable activities with his wife, visit the senior center	His wife’s told him whe was planning to leave him
C5	66	M	Retired	Exercise, wash windows, do archery	Girlfriend passed away a year ago
C6	81	Human	Teacher	Engage in enjoyable activities (weight watchers meetings, walking), reconnecting with family and neighbors, conflict resolution, get Christmas gifts	Feeling tired, feeling isolated, having knee surgery, moving with her child and grandchildren
C7	62	Male	Retired trooper, social worker	Reconnect with friends Has multiple health issues and severe injuries that forced him to retire.	Feels isolated from old friends. Is embarrassed for not being the main income earner in-home
C8	64	Female	n/a	Exercise, do pleasurable activities with husband, clean house	Loss of son 10 years ago, living sons are not talking to her, husband is hoarding things
C9	68	Female	Teacher/tutor	Do pleasurable activities, communicate with son, recognize how activities made her feel	House got damaged resulted in high expenses, had a traumatic car accident
C10	67	Male	Retired	Do pleasurable activities, resolve how to manage wife’s illness	Progression of wife’s disease impacts his daily routine.
C11	65	Male	Residential Counselor Supervisor	Manage guilt around family’s death, address intimacy problems, declutter room	Feeling guilt about deceased family members

**Table 2.** Processes used by the clients and therapists to inform goal identification, the challenge each process addresses, and how.

Process of goal setting	Challenge in this process	How challenge is addressed
Select goal or problem	It is difficult to know which goal to address among multiple existing problems	Accounting for the multitude of problems across different aspects of life (controllable, aligned with values) Revisiting unresolved past problems and goals (revisit avoided goals, readiness to move on) Anticipating future challenging situations
Simplify complex goal to specific one	The problem is too broad to be addressed Not knowing what goal could be helpful	Understanding different aspects of problems - Understanding how problems manifest - Breaking down problems - Reinterpret problems Identifying feasible goals
Adjust goal	The goal is not achievable or no longer fits client needs	Understanding client skills and learning new skills Anticipating and reflecting on barriers Reinterpreting current goals and practices
